# Impact of Metabolic Surgery on Gut Microbiota and Sera Metabolomic Patterns among Patients with Diabetes

**DOI:** 10.3390/ijms23147797

**Published:** 2022-07-14

**Authors:** Hsien-Hao Huang, Tzu-Lung Lin, Wei-Jei Lee, Shu-Chun Chen, Wei-Fan Lai, Chia-Chen Lu, Hsin-Chih Lai, Chih-Yen Chen

**Affiliations:** 1 Department of Emergency Medicine, Taipei Veterans General Hospital, Taipei 11217, Taiwan; hhhuang@vghtpe.gov.tw; 2 Institute of Emergency and Critical Medicine, National Yang Ming Chiao Tung University College of Medicine, Taipei 11221, Taiwan; 3 Department of Medical Biotechnology and Laboratory Science, College of Medicine, Chang Gung University, Taoyuan 33302, Taiwan; f87445101@ntu.edu.tw; 4 Microbiota Research Center and Emerging Viral Infections Research Center, Chang Gung University, Taoyuan 33302, Taiwan; 5 Department of Surgery, Min-Sheng General Hospital, Taoyuan 33044, Taiwan; wjlee_obessurg_tw@yahoo.com.tw; 6 Taiwan Society for Metabolic and Bariatric Surgery, Taipei 11031, Taiwan; n002916@e-ms.com.tw; 7 Department of Nursing, Chang-Gung Institute of Technology, Taoyuan 33303, Taiwan; 8 Department of Medicine, College of Medicine, Chang Gung University, Taoyuan 33302, Taiwan; f84032411@gmail.com; 9 Department of Chest Medicine, Internal Medicine, Fu Jen Catholic University Hospital, Fu Jen Catholic University, New Taipei City 24352, Taiwan; chiachen.lulu@gmail.com; 10 Department of Respiratory Therapy, Fu Jen Catholic University, New Taipei City 24205, Taiwan; 11 Research Center for Chinese Herbal Medicine and Research Center for Food and Cosmetic Safety, College of Human Ecology, Chang Gung University of Science and Technology, Taoyuan 33303, Taiwan; 12 Department of Laboratory Medicine, Linkou Chang Gung Memorial Hospital, Taoyuan 33305, Taiwan; 13 Central Research Laboratory, Xiamen Chang Gung Allergology Consortium, Xiamen Chang Gung Hospital, Xiamen 361028, China; 14 Division of Gastroenterology and Hepatology, Department of Medicine, Taipei Veterans General Hospital, Taipei 11217, Taiwan; 15 Faculty of Medicine, College of Medicine, National Yang Ming Chiao Tung University, Taipei 11221, Taiwan; 16 Bariatric and Metabolic Surgery Center, Taipei Veterans General Hospital, Taipei 11217, Taiwan; 17 Chinese Taipei Society for the Study of Obesity, Taipei 11031, Taiwan; 18 Taiwan Association for the Study of Small Intestinal Diseases, Taoyuan 333423, Taiwan

**Keywords:** metabolic surgery, metabolomics, microbiota, obesity, type 2 diabetes mellitus

## Abstract

Metabolic surgery is a promising treatment for obese individuals with type 2 diabetes mellitus (T2DM), but the mechanism is not completely understood. Current understanding of the underlying ameliorative mechanisms relies on alterations in parameters related to the gastrointestinal hormones, biochemistry, energy absorption, the relative composition of the gut microbiota, and sera metabolites. A total of 13 patients with obesity and T2DM undergoing metabolic surgery treatments were recruited. Systematic changes of critical parameters and the effects and markers after metabolic surgery, in a longitudinal manner (before surgery and three, twelve, and twenty-four months after surgery) were measured. The metabolomics pattern, gut microbiota composition, together with the hormonal and biochemical characterizations, were analyzed. Body weight, body mass index, total cholesterol, triglyceride, fasting glucose level, C-peptide, HbA1c, HOMA-IR, gamma-glutamyltransferase, and des-acyl ghrelin were significantly reduced two years after metabolic surgery. These were closely associated with the changes of sera metabolomics and gut microbiota. Significant negative associations were found between the *Eubacterium eligens* group and lacosamide glucuronide, UDP-L-arabinose, lanceotoxin A, pipercyclobutanamide B, and hordatine B. Negative associations were identified between *Ruminococcaceae* UCG-003 and orotidine, and glucose. A positive correlation was found between *Enterococcus* and glutamic acid, and vindoline. Metabolic surgery showed positive effects on the amelioration of diabetes and metabolic syndromes, which were closely associated with the change of sera metabolomics, the gut microbiota, and other disease-related parameters.

## 1. Introduction

Diabetes and related metabolic syndromes are major public health problems [1]. Globally, an estimated 422 million adults were living with diabetes in 2014, compared to 108 million in 1980. The increasing prevalence, variable pathogenesis, progressive natural history, and complications of diabetes emphasize the urgent need for effective treatment strategies. At present, metabolic surgery is reckoned as the most effective treatment option to improve glucose control and long-term complications associated with hyperglycemia in patients with obesity [2].

The gut–brain axis is considered one of the major regulators to modulate the glucose homeostasis. The enteric glucose condition was detected by gut endocrine cells to secret intestinal neuro-hormones, such as glucagon-like peptide-1 (GLP-1), 5-hydroxytryptamine or apelin [3,4,5,6,7,8]. These gut hormones activate enteric neurons and transmit neuronal messages through vagal afferent pathways to modulate glycemia regulation and improve diabetic conditions through the gut–brain axis [4,7,9]. Furthermore, gut microbiota dysbiosis induces GLP-1 resistance [4] and modulates metabolites to regulate serotonin biosynthesis [6]. The concept of the microbiota–gut–brain axis was recently proposed and is increasingly acceptable. A dysregulated microbiota–gut–brain axis can affect the host’S behavior [10,11,12] and irritable bowel syndrome [13,14]. Thus microbiota, metabolites, and gut hormones might be involved in microbiota–gut–brain signaling and initiate the solutions for diabetes and metabolic syndrome.

Although the mechanisms behind these ameliorative metabolic effects remain only partially understood, current knowledge on these complex mechanisms indicate that the metabolic and immune effects of metabolic surgery might mainly be due to the deviation of nutrients, calorie restriction, reduced amounts of adipose tissue, and weight reduction. Detailed analyses further highlights that the altered gastrointestinal release of gut hormones related to carbohydrate metabolism (the rapid and continuous release of insulin), appetite and the degree of satiety [GLP-1, peptide YY (PYY), ghrelin] [15], altered the gut microbiota [16,17,18]. The change of metabolomics patterns [19,20,21] also seems to play important roles in reducing inflammation and improving insulin sensitivity. From a clinical perspective, these changes are associated with the remission of diabetes in patients with obesity and type 2 diabetes mellitus (T2DM), the prevention of diabetes in patients with insulin resistance without overt T2DM, and the prevention of both microvascular and macrovascular complications for patients with morbid obesity [2].

In the present study, we focused on characterizing patients with diabetes mellitus and related syndromes and evaluated the effects and markers of metabolic surgery operations in a longitudinal (before surgery and three, twelve, and twenty-four months after surgery) manner. The metabolomics pattern, gut microbiota composition, together with the hormonal and biochemical characterizations, were analyzed.

## 2. Results

### 2.1. Changes in Clinical and Circulating Biomarkers after Metabolic Surgery

In total, 13 T2DM patients (age: 42.4 ± 8.6 years, eight males and five females) undergoing metabolic surgery were enrolled. Six patients underwent laparoscopic gastric bypass (GB), four patients underwent laparoscopic sleeve gastrectomy (SG), and three patients underwent laparoscopic duodeno-jejunal bypass with sleeve gastrectomy (DJB–SG). Participant characteristics and changes in the effect of metabolic surgery on the clinical and circulating metabolic biomarkers in patients are shown in Table 1. No trends of differences in these parameters were observed among the GB (*n* = 6), SG (*n* = 4), and DJB–SG (*n* = 3) groups. Body weight, BMI, total cholesterol, triglyceride, fasting glucose level, C-peptide, HbA1c, HOMA-IR, gamma-glutamyltransferase, and des-acyl ghrelin were significantly reduced after metabolic surgery. Furthermore, the levels of high-density lipoprotein cholesterol were increased.

### 2.2. Changes in the Gut Microbiota after Metabolic Surgery

**Figure 1:**
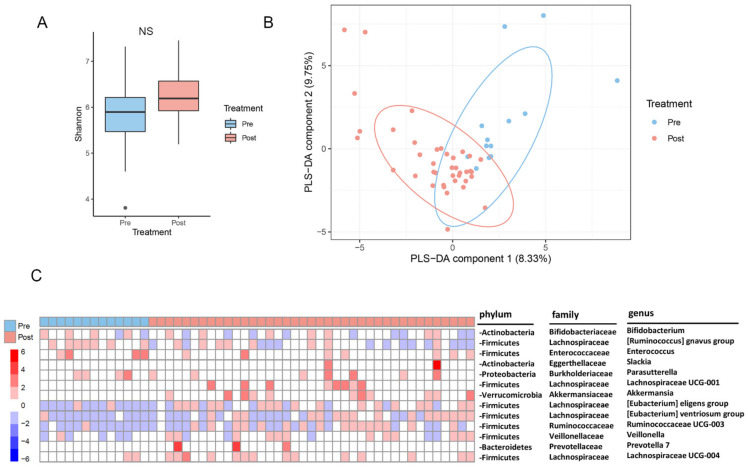
Changes in the gut microbiota after metabolic surgery. (**A**) Pre-surgery and post-surgery alpha diversity. Shannon’s index between pre-surgery and post-surgery is shown (*p* = 0.0987). While there is a trend towards a higher alpha diversity post-surgically, this was not statistically significant. NS, not significant. (**B**) Pre-surgery and post-surgery beta diversity. Pre-surgical and post-surgical partial least squares discriminant analysis (PLS-DA) score plot is shown. Differential pre-surgical and post-surgical clustering is observed. (**C**) Differential bacterial composition after metabolic surgery. The bacterial taxa between pre-surgery and post-surgery are compared by using DEseq2. Heat map (converting to z-scores of the rows) of the differential bacterial genus abundance after surgery are shown (adjusted *p* < 0.05, |log2 fold change| > 1.5).

The gut microbiota composition is reckoned to be closely associated with the development of diabetes and related metabolic syndromes. Next, we characterized the change in the gut microbiota composition after metabolic surgery. The richness of the gut microbiota, indicated by the Shannon index pre-surgically and post-surgically, is shown in Figure 1A. The overall change in the alpha diversity indicates a trend in the increase after metabolic surgery, though this is not statistically significant (*p* = 0.0987) (Figure 1A). By contrast, the overall beta diversity analysis shown as the PLS-DA components indicate a significant variation before and after metabolic surgery (Figure 1B). Subsequent comparative analysis of bacterial genus between pre-surgery and post-surgery indicates that the relative abundances of bacterial microbiota were significantly changed after metabolic surgery (Figure 1C). Among these, *Lachnospiraceae* UCG-001 and UCG-004, the *Ruminococcaceae* UCG-003 group, the *Eubacterium eligens* group, *Eubacterium ventriosum* group, and Veillonella belonging to the phylum Firmicutes, Prevotella 7 belonging to Bacteroidetes, and Slackia (Actinobacteria) increased in abundance after metabolic surgery. Furthermore, Akkermansia belonging to Verrucomicrobia also increased after metabolic surgery. By contrast, the abundance of some bacteria was decreased in comparison to those measured in the pre-surgery group. These included *Bifidobacterium* (Actinobacteria), the *Ruminococcus gnavus* group, *Parasutterella* (Proteobacteria), and *Enterococcus* (Figure 1C). Overall, these results suggest that metabolic surgery modifies the diversity and composition of some gut bacterial microbiota.

### 2.3. Changes in the Serum Metabolites after Metabolic Surgery

**Figure 2:**
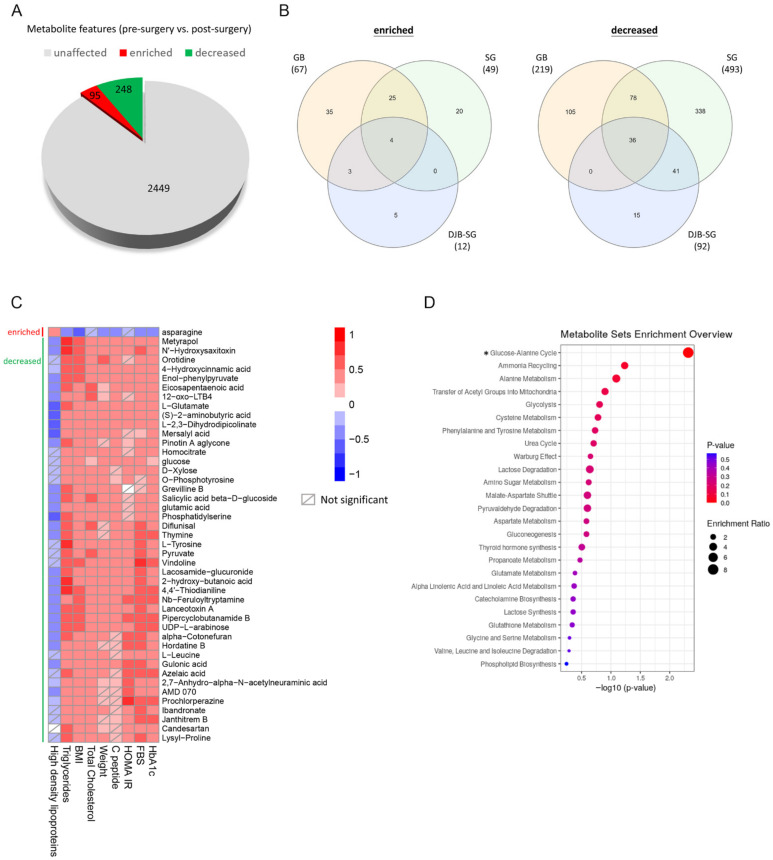
Changes in the serum metabolites after metabolic surgery. (**A**) Differential expressed metabolite features after surgery. Pre-surgery and post-surgery sera metabolite features identified by using an untargeted metabolomic platform gas chromatography-mass spectrometry (GC-MS) and liquid chromatography-mass spectrometry (LC-MS) were annotated and compared. After metabolic surgery, 95 annotated metabolite features were enriched, and 248 features were decreased (adjusted *p* < 0.05). (**B**) Venn diagrams showed the numbers of common and unique metabolites altered in patients after having three different metabolic surgery techniques (GB, laparoscopic gastric bypass; SG, laparoscopic sleeve gastrectomy; DJB–SG, laparoscopic duodenal-jejunal bypass with sleeve gastrectomy). (**C**) Correlation between differential-expressed metabolites and clinical indices. The correlation between 207 differential-expressed metabolites with KEGG ID, and 9 obesity and diabetes mellitus-associated clinical indices were analyzed. Forty-five metabolites (one enriched and forty-four decreased) were significantly correlated with at least seven clinical indices (*p* < 0.05). Heat map of the Spearman’s rank correlation coefficient between these forty-five metabolites and nine clinical indices is shown. (**D**) Pathway enrichment analysis of the clinical indices-correlated metabolites. The pathway enrichment analysis of 45 clinical indices-correlated metabolites was performed by using pathway-associated metabolite sets (SMPDB), which contains 99 metabolite sets based on normal human metabolic pathways on the MetaboAnalyst 4.0. (https://www.metaboanalyst.ca/ (accessed on 11 August 2020)). *, *p* < 0.05.

The sera metabolomics compositions have previously been reported to change after metabolic surgery. To address whether any metabolites were significantly changed after metabolic surgery, sera of the 13 patients were collected in a longitudinal manner (before metabolic surgery and three, twelve, and twenty-four months after metabolic surgery) and subjected to GC-MS and LC-MS untargeted metabolomics analysis. As shown in Figure 2, among the 2792 metabolites analyzed, 95 were significantly increased, and 248 were significantly decreased in concentrations after metabolic surgery (Figure 2A). The methods of surgery might have different impacts on metabolome. Therefore, the changes of metabolites in sera of patients undergoing three different surgical techniques were also analyzed. Venn diagrams indicate the numbers of common and unique metabolites altered in patients after having three different metabolic surgery techniques (GB, SG or DJB–SG) (Figure 2B). The highest numbers of commonly changed metabolites were observed between GB and SG groups. In addition, approximately 84% of decreased metabolites in the DJB–SG group were the same as those in SG group (Figure 2B).

As the change of sera metabolites may be closely associated with the development of diabetes and related syndromes, concentrations of these metabolites were subject to analysis to see their correlation with the related nine clinical indices, which were significantly different before and after metabolic surgery. As shown in Figure 2C, a total of forty-five metabolites, with one being increased and forty-four decreased, whose concentrations were statistically associated with more than seven out of the nine clinical indices were highlighted. Among these, reduction in the concentrations of 44 metabolites were related to reduced levels of HbA1c, fasting blood sugar, HOMA-IR, C-peptide, body weight, total cholesterol, BMI, and triglyceride. By contrast, concentrations of these metabolites were negatively associated with that of high-density lipoprotein cholesterol.

The 45 metabolites may be involved in some biochemical pathways related to diabetes and glucose control. To verify this, they were subject to functional biochemical pathways in the MetaboAnalyst 4.0 (https://www.metaboanalyst.ca/ (accessed on 11 August 2020)). The results in Figure 2D indicate that only the glucose–alanine cycle pathway was significantly activated after metabolic surgery in contrast to the pre-surgical status. Relatively, pathways, such as ammonia recycling, alanine metabolism, glucose metabolism, etc., also showed a trend in activation, although statistics did not show significance.

### 2.4. Changes of Branched Chain Amino Acids (BCAA) after Metabolic Surgery

**Figure 3:**
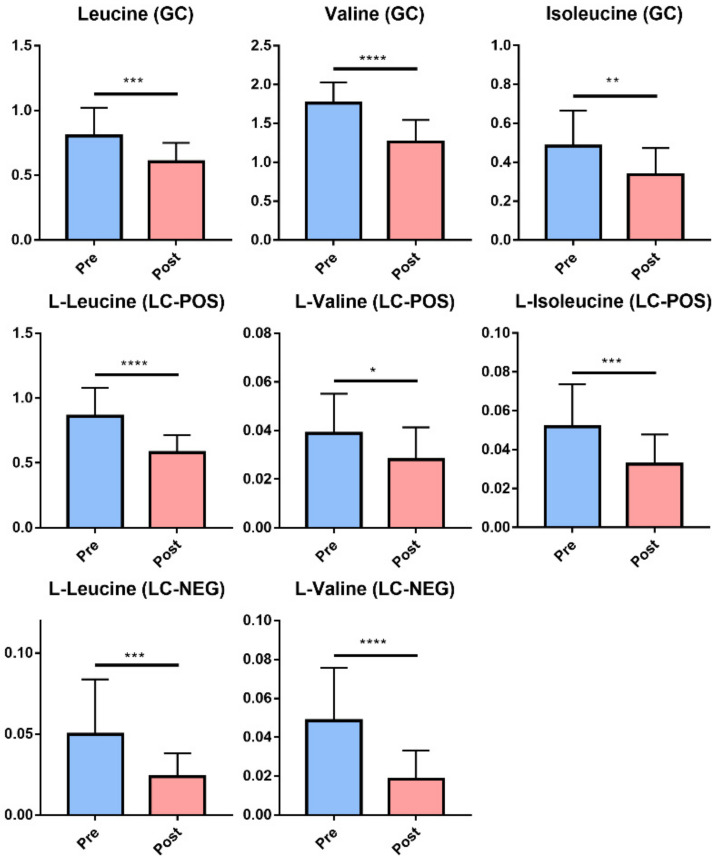
Changes of Branched Chain Amino Acids (BCAA) after metabolic surgery. The changes of BCAA (Leucine, Valine, and Isoleucine) in GC-MS (upper panel) and LC-MS (LC-POS, LC-MS positive ion mode, middle panel; LC-NEG, LC-MS negative ion mode, bottom panel) untargeted metabolomics analysis after metabolic surgery were shown. Data were presented as the mean ± SD. *, *p* < 0.05; **, *p* < 0.01; ***, *p* < 0.001; ****, *p* < 0.0001 (unpaired Student’s *t*-test).

It is well documented that the increase of BCAA levels in obese individuals is associated with a degradation of insulin sensitivity [22,23]. Accordingly, the changes of BCAA (Leucine, Valine, and Isoleucine) in gas chromatography-mass spectrometry (GC-MS) and liquid chromatography-mass spectrometry (LC-MS) untargeted metabolomics analysis were specifically surveyed. Significantly decreased levels of BCAA after metabolic surgery were observed (Figure 3). In addition, the reduction in the level of leucine was related to reduced levels of HbA1C, FBS, HOMA IR, C-peptide, body weight, total cholesterol, BMI, and triglyceride, whereas it was negatively associated with that of high-density lipoprotein (Figure 2C).

### 2.5. Association of Changes between the Gut Microbiota and Sera Metabolites after Metabolic Surgery

**Figure 4:**
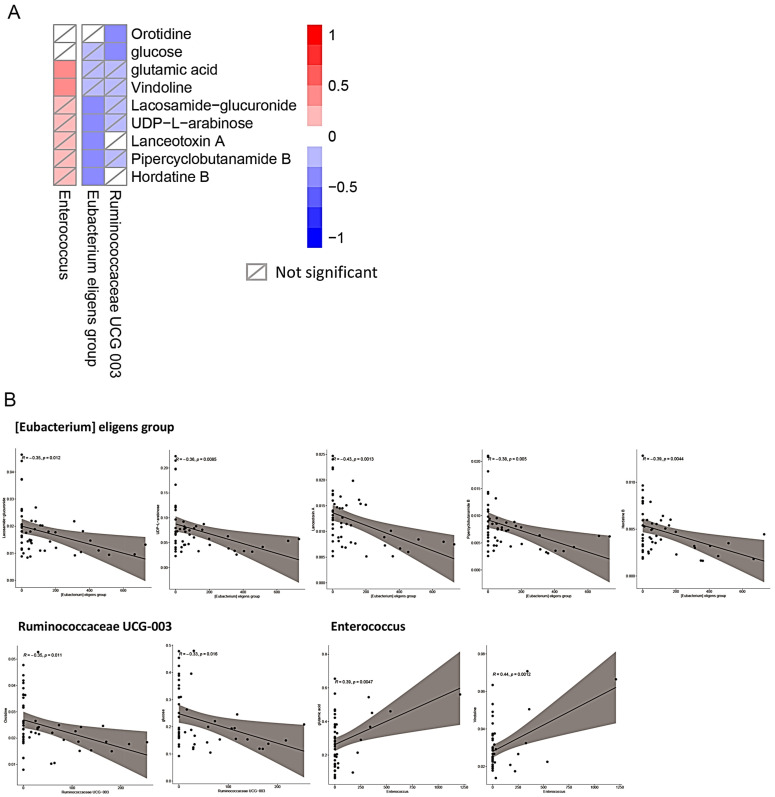
Association of changes between the gut microbiota and sera metabolites after metabolic surgery. (**A**) Correlation between the differential bacterial genus and the clinical indices-correlated metabolites. The correlation between 45 clinical indices-correlated metabolites and the 13 differential bacterial genera were analyzed, and the heat map of the Spearman’s rank correlation coefficient (*p* < 0.02) are shown. (**B**) Scatterplots between the metabolites and bacterial genus. Scatterplot with a line of best fit illustrates the relationship between the metabolites and bacterial genus, which reveals a significant correlation in (**A**).

The change of sera metabolites may be related to the change in certain compositions of the gut microbiota after metabolic surgery. To verify if there is any potential relationship between the changed metabolites and gut microbiota, Spearman’s correlation analysis was performed for association analysis. The results obtained indicate that the reduction in nine metabolites was closely associated with three microbiota groups, namely, *Enterococcus*, the *Eubacterium* eligens group, and *Ruminococcaceae* UCG-003 (Figure 4A). Among these, significant negative associations were found between the *Eubacterium eligens* group and lacosamide glucuronide (*p* = 0.012), UDP-L-arabinose (*p* = 0.0085), lanceotoxin A (*p* = 0.0013), pipercyclobutanamide B (*p* = 0.005), and hordatine B (*p* = 0.0044) (Figure 4B). Negative associations were also identified between *Ruminococcaceae* UCG-003 and orotidine (*p* = 0.011), and glucose (*p* = 0.016). By contrast, a positive correlation was found between *Enterococcus* and glutamic acid (*p* = 0.0047), and vindoline (*p* = 0.0012).

## 3. Discussion

Our results show that metabolic surgery approaches significantly improves clinical syndromes. These are closely associated with the change of sera metabolomics and the gut microbiota pattern. Among these, the abundance of *Enterococcus, Eubacterium eligens* group, and *Ruminococcaceae* UCG-003 were closely related to a total of nine sera metabolites that were associated with the glucose–alanine cycle activity and the progress of disease amelioration. Metabolic surgery showed positive effects on the amelioration of diabetes and metabolic syndromes, which are closely associated with the change of sera metabolomics, the gut microbiota, and other disease-related parameters.

To achieve amelioration of diabetes and its associated consequences, strategies, such as eating a healthy diet, enhanced physical activities, prescription of medications, regular screening, or treatment for complications, etc. can be considered (https://www.afro.who.int/health-topics/diabetes (accessed on 25 August 2020)). On the other hand, a healthy lifestyle, including a healthy diet and maintaining optimal body weight, is also beneficial in preventing or delaying the onset of T2DM [24]. Currently, metabolic surgery is characterized as being a safe, highly-effective, and long-lasting treatment approach for patients with diabetes, obesity, and its associated co-morbidities [25,26]. Due to the obesity pandemic, metabolic surgery is currently the second-most frequent intra-abdominal procedure. Therefore, gastroenterologists and surgeons have to understand the multiple physical and physiological changes due to anatomic reconfiguration following metabolic surgery. Among the underlying mechanisms, besides the loss of weight and adipose tissue, metabolic surgery generally results in the increased release of gut hormones related to carbohydrate metabolism, appetite control, and the degree of satiety [27,28]. On the other hand, the changes in sera metabolites and gut microbiota were also observed [15]. Thus, metabolic surgery not only deals with the removal of gastric or intestinal tissues, or changes in their relative bypasses but also various other ameliorative functions. These included a change in the biochemical, metabolic, and physiological activities in patients to achieve the amelioration of diabetes and related syndromes [2,29]. Among these factors, the metabolic activities related to carbohydrate metabolism seemed to be most significantly affected. Therefore, bariatric surgery is also frequently reckoned as metabolic surgery in the aspect of functional amelioration. Besides reducing patients’ mortality, metabolic surgery also decreases the risks associated with many other comorbidities, such as cardiovascular disorders, chronic kidney diseases, albuminuria, etc. [30].

Changes in the levels of some diabetes and obesity-related hormones are observed in this study, though some of them do not show statistical significance before and after metabolic surgery. For example, levels of GLP-1, PYY, and FGF-19 increased, while GIP decreased after metabolic surgery (Table 1). These phenomena are basically in concordance with many previous reports based on the use of the meta-analysis approach [15,29]. On the other hand, while concentrations of both acyl ghrelin and des-acyl ghrelin showed a trend of reduction after metabolic surgery, only the levels of des-acyl ghrelin showed statistical significance in this study (Table 1). Ghrelin incipiently encoded by the preproghrelin gene was reported to be involved in the regulation of secretion of the growth hormone, energy homeostasis, and glucose metabolism [15,27]. After post-translational processing, two ghrelin circulating isoforms, acyl ghrelin and des-acyl ghrelin, showing distinct metabolic effects were formed, respectively [27,31,32]. Specifically, des-acyl ghrelin existed as the predominant isoform of ghrelin in the circulation and involves multiple functions, such as the control of cell growth, tissue injury, and glucose and lipid metabolism [31]. Although a more in-depth study is necessary in characterizing acyl ghrelin/des-acyl ghrelin functions, results obtained in this study highlight the significant effect of metabolic surgery on the reduction of ghrelin. Another phenomenon observed is that while the levels of fasting glucose were significantly reduced by all metabolic surgery procedures, those of fasting insulin did not show a statistically significant difference, even though a clear trend of reduction was observed after metabolic surgery (Table 1). As we followed the original measurements and the raw data were not modified before the statistical analysis, these results might be due to the variations detected from the patients’ clinical samples. Additionally, in terms of the liver function test, the level of gamma-glutamyltransferase was reduced by metabolic surgery, though the results were not significantly affected (Table 1). The previous study also indicates a significant correlation between the levels of gamma-glutamyltransferase and BMI, HbA1c, cholesterol, and triglyceride [33]. Therefore, results obtained may still suggest that the restoring effects after metabolic surgery may involve gamma-glutamyltransferase-associated functions and phenotypes. On the other hand, in terms of the concentration of total bile acids (μM), the previous study reported that an altered bile acid metabolism was observed after metabolic surgery [21]. Moreover, accumulating pieces of evidence indicate that bile acids play critical roles in metabolic regulation as signaling molecules, mostly mediated through the nuclear receptor FXR and membrane receptor TGR5 [21,34]. Therefore, bile acids appear to be involved in controlling important physiological activities after metabolic surgery. Although we did not detect a significant variation in the total bile acids concentration in this study, it is highly possible that the composition of bile acids might vary, which needs more clinical case numbers.

Among the many different approaches for metabolic surgery, our previous studies highlighted the advantages of DJB–SG [35]. In this study, due to a limited number of patients undergoing metabolic surgery, we could not proceed into statistical analysis focusing on the benefits derived from DJB–SG, in contrast to many other approaches for metabolic surgery. However, after DJB–SG, one advantage is that patients could still be subject to gastroscopy, in contrast to the other approaches wherein it became relatively more difficult. Therefore, DJB–SG might be more suitable for patients living in areas with a high prevalence of gastric cancer, such as Asia [35].

In contrast to the normal population, the change in the microbiota (dysbiosis) and metabolomics patterns was shown to play a critical role in the development of diabetes and obesity-related comorbidities. By contrast, management of the gut microbiota composition is also reported to play a vital role in the ameliorative efficacy of patients that received metabolic surgery [36,37]. Results obtained in this study also indicate a change in the gut microbiota structure after metabolic surgery (Figure 1), which was basically in concordance with those reported from previous studies [37]. Among the changes in the gut microbiota structure, some essential bacterial microbiotas were identified to have a close relationship with diabetes and the related indices. Both alpha diversity and beta diversity varied after metabolic surgery, though a trend was observed only for alpha diversity (Figure 1). Among these, an abundance of bacterial groups classified in the phyla of Firmicutes, Bacteroidetes, Actinobacteria, Proteobacteria, and Verrucomicrobia either increased (*Lachnospiraceae* UCG-001 and UCG-004, the *Ruminococcaceae* UCG-003 group, *Eubacterium eligens* group, the *Eubacterium ventriosum* group, Veillonella, Prevotella 7, Slackia, and Akkermansia) or decreased (Bifidobacterium, the *Ruminococcus gnavus* group, *Enterococcus*, and *Parasutterella*) after metabolic surgery. Akkermansia has previously shown to be closely related to the anti-obesity effect and amelioration of diabetes [38]. Besides, results shown from many previous studies also indicate that an abundance of some bacteria varied in this study are also related to the amelioration of diabetes and related diseases. For example, in a previous study that highlighted the therapeutic efficacy of carrot juice fermented with *Lactobacillus* rhamnosus GG (LGG) on T2DM, an abundance of some gut bacterial microbiota, such as the *Lachnospiraceae* NK4A136 group and Akkermansia, were increased [39]. On the other hand, the administration of Xiexin Tang, a traditional Chinese medicinal formula that has been clinically used to treat diabetes for thousands of years, also improved the syndromes in type 2 diabetic rats [40]. Further studies indicate that *Lachnospiraceae* UCG-001 and the *Prevotellaceae* NK3B31 group also increased in their relative abundance [40]. Another example came from the ameliorative effects of 1-deoxynojirimycin (DNJ) that exerts hypoglycemic effects. DNJ treatment relieved gut dysbiosis in diabetic mice and promoted the growth of Lactobacillus, the *Lachnospiraceae* NK4A136 group, and *no-rank Lachnospiraceae* (*p* < 0.05), and suppressed the growth of *Ruminococcaceae* UCG-014, *Ruminococcus* (*p* < 0.05) [41]. Concordantly, the abundance of *Lachnospiraceae* UCG-005 was significantly higher in the stomachs of the wild type Wistar rats, in contrast to the Goto-Kakizaki (GK) rat that was developed from repeated inbreeding of the glucose-intolerant Wistar rats [42].

After systematic bioinformatics analyses, among the 45 significantly changed metabolites detected after metabolic surgery, the glucose–alanine cycle was highlighted (Figure 2). This cycle, also known as the Cahill cycle, is a series of reactions in which amino groups and carbons from the muscle are transported to the liver [43]. When muscles degrade amino acids for energy needs, the resulting nitrogen is converted to pyruvate via transamination to form alanine. This reaction is achieved by the enzyme alanine aminotransferase, which converts L-glutamate and pyruvate into α-ketoglutarate and L-alanine [43]. The resulting L-alanine is shuttled to the liver where nitrogen enters the urea cycle and pyruvate is used to make glucose [44]. The increased activity of the glucose–alanine cycle may indicate the enhanced exchange of metabolites between the secretion of pyruvate-derived alanine or lactate in the muscle into the liver, and the release of glucose from the liver into the muscle. The process is mediated by systematic blood circulation for the efficient exchange of metabolites. As the glucose produced in the liver is transferred to the muscle more efficiently after metabolic surgery, this might be an underlying mechanism by which the blood glucose concentration is significantly reduced. On the other hand, the glucose transferred to the muscle cells was hydrolyzed to form pyruvate accompanied by the release of adenosine triphosphate (ATP). The reduced glucose concentration might be related to an increase in the ATP formation, which might be converted to heat release [45]. Subsequent mechanistic studies are warranted. Besides the glucose–alanine cycle, the activities of other biochemical metabolomics pathways related to this cycle, such as ammonia recycling, alanine metabolism, transfer of the acetyl group into mitochondria, glycolysis reactions, etc., were also enhanced in the same trend (Figure 2). Together, these may suggest an increased efficacy on the consumption of blood glucose after metabolic surgery. How and why such a phenomenon was achieved by metabolic surgery remains unclear, and this may need a more in-depth study.

While patients receiving metabolic surgery showed ameliorative effects in this study, changes in the gut microbiota composition and the metabolomics pattern after metabolic surgery were closely associated with the development of the symptoms. A detailed association study indicates that the abundance of three microbiota groups is closely associated with sera metabolic marker concentrations that are closely related to the ameliorative effects of metabolic surgery (Figure 4). Among these, significant negative associations were identified between the *Eubacterium eligens* group and lacosamide glucuronide, UDP-L-arabinose, lanceotoxin A, pipercyclobutanamide B, and hordatine B (Figure 4A). Similarly, negative associations were also identified between *Ruminococcaceae* UCG-003 and orotidine, and glucose. By contrast, a positive correlation was found between *Enterococcus* and glutamic acid, and vindoline. These results indicate that an increased amount of the *Eubacterium eligens* group and *Ruminococcaceae* UCG-003, and a decreased amount of *Enterococcus* may be associated with the ameliorative effects of metabolic surgery. As the bacterial species and strains classified in the three groups were diverse, more studies should be performed to further isolate and characterize the consortium of bacteria that might be involved in such metabolic activities.

Our study has some limitations. First, the number of T2DM patients with obesity during a 2-year follow-up after metabolic surgery was only 13. However, these results were enough to show that beta-diversity analysis and the subsequent comparative analysis of bacterial genus had a significant change before and 2 years after metabolic surgery. Second, the enrolled numbers in different types of metabolic surgery were small (GB, *n* = 6; SB, *n* = 4; DJB–SG, *n* = 3). Additionally, the significant changes in gut microbiome composition and sera metabolites, as well as the significant associations between metabolites and ameliorative effects 2 years after metabolic surgery, were identified. The results need further confirmations in different types of metabolic surgery by using a larger sample size. The causal effect also needs further investigation.

## 4. Materials and Methods

### 4.1. Patients and Metabolic Surgery

A hospital-based design was adapted for the present study. Patients with T2DM who had undergone a gastric bypass (GB), sleeve gastrectomy (SG), or laparoscopic duodeno-jejunal bypass with sleeve gastrectomy (DJB–SG) procedures were enrolled in our study. The diagnostic and inclusive criteria were as follows: <1> T2DM discovered more than six months with HbA1c 8%, under the close monitoring and medical treatment, including diet control, oral anti-diabetic agents, or insulin from an endocrinologist; <2> body mass index (BMI) between 27.5 kg/m^2^ and 35 kg/m^2^; <3> willingness to receive additional therapy with diet control and exercise; <4> willingness to accept follow-up consultations; and <5> willingness to sign an informed consent.

Candidates were excluded if they: <1> had experienced cancer within the last 5 years; <2> had human immunodeficiency virus or active pulmonary tuberculosis; <3> had experienced cardiovascular diseases or cardiovascular instability within the last six months; <4> had a pulmonary embolism or uncontrolled coagulopathy; <5> had serum creatinine > 2.0 mg/dL; <6> had chronic hepatitis B, hepatitis C, liver cirrhosis, or inflammatory bowel diseases; <7> had acromegaly or had received an organ transplantation; <8> had undergone any other metabolic surgery or gastrointestinal surgery other than cholecystectomy, or had prior abdominal septicemia; <9> were an alcoholic or abused drugs, had psychiatric diseases; and <10> had other uncooperative conditions.

Finally, six patients undergoing GB, four patients undergoing SG, and three patients undergoing DJB–SG were enrolled in this prospective, longitudinal study. The treatment decision was based on the clinical background of individual subjects.

### 4.2. Surgical Technique

#### 4.2.1. Laparoscopic Gastric Bypass (GB)

Laparoscopic GB led to a digestive tract that bypasses the foregut, especially the duodenum and a thin, tube-like remnant stomach. The procedure was reported to result in the encouragement weight loss and resolution of T2DM and metabolic syndrome [46]. Similar to the techniques reported in our previous study, a 5-trocar laparoscopic technique was conducted, and then a gastric tube approximately 2 cm wide was created along the lesser curvature. A Billroth II procedure, followed by the 120 cm small bowel distal to the ligament of Treitz, was then performed. No drainage tube was left after the surgery.

#### 4.2.2. Laparoscopic Sleeve Gastrectomy (SG)

SG results in the reduction of the stomach. Under the standard 5-trocar laparoscopic technique, the pylorus was identified, and the greater curvature, which included the whole fundus, was resected from the distal antrum to the angle of His with the transaction of the stomach, which was about 2 cm wide along the lesser curvature. The procedure culminated in a tube-like stomach. The resected portion of the stomach was extracted from the periumbilical trocar site. A running absorbable suture was applied to reinforce the staple line to reduce the risk of hemorrhage and leakage [47].

#### 4.2.3. Laparoscopic Duodenojejunal Bypass with Sleeve Gastrectomy (DJB–SG)

The procedure was performed in the “French” position (surgeon between the patient’s legs) through a standard 5-trocar laparoscopic technique. The entire greater curvature was mobilized from the omentum. A large portion of the stomach was resected from the distal antrum. The staple line was routinely reinforced with a running unabsorbable suture. The duodenum was dissected free of the lower and posterior wall. After the ligament of Treitz was identified, the length of the alimentary limb of the small bowel was decided according to the BMI, with a 150 cm biliopancreatic limb for patients whose BMI was <35 kg/m^2^. The selected loop was ascended antecolically without dividing the omentum. A side-to-side duodenojejunal anastomosis was created. The stapler defect was closed with a two-layer absorbable running suture. At the end of the surgery, we performed an air-leak test and left a drain [48].

### 4.3. Study Protocol and Anthropometric Measurement

Four separate occasions of follow-up occurred for all participants: at the baseline (before surgery), and twelve, and twenty-four months postoperatively. Routine laboratory tests, including serum total cholesterol, triglyceride, high-density lipoprotein cholesterol, low-density lipoprotein cholesterol, fasting blood sugar, HbA1c, and C-peptide, as well as anthropometric measurements, were performed on each study day. The homeostatic model assessment (HOMA-IR) index, calculated according to the formula plasma glucose (mmol/L) × insulin (μU/mL)/22.5, was measured and represented insulin resistance [49].

### 4.4. DNA Extraction from Human Stool Samples

Bacterial DNA was extracted and purified by previously published methods with slight modifications [50]. About 100 mg of stool sample was washed thrice with PBS and re-suspended in the extraction buffer (100 mM Tris-HCl, 40 mM EDTA, 1% SDS; pH 9.0) with 0.3 g of glass beads (0.1 mm in diameter) and 500 μL of buffer-saturated phenol. The mixture was vigorously shaken for 30 s in the FastPrep FP120 homogenizer (Q-Biogene, Irvine, CA, USA) at a speed of 5.0 m/s. The sample was then centrifuged at 12,000 *g* for 5 min. After centrifugation, 400 μL of the supernatant was added into an equal amount of phenol-chloroform-isoamyl alcohol (25:24:1, v/v). The mixture was shaken again in the FastPrep FP120 at a speed of 4.0 m/s for 45 s then centrifuged again at 12,000 *g* for 5 min. The DNA was precipitated with 3M sodium acetate (pH 5.4) and isopropanol. The DNA was then air-dried and dissolved in TE buffer (10 mM Tris-HCl, 1 mM EDTA, pH 8.0). The concentration of DNA was adjusted to 10 ng/μL, and the samples were stored at −20 °C.

### 4.5. 16S rDNA-Based Metagenomics Analysis Pipeline

We used a protocol modified from our previous studies [18,51,52]. The alpha and beta diversity were measured by QIIME2 according to the Shannon index and Bioconductor R package “mixOmics” (version 6.12.1) with partial least squares-discriminant analysis (PLS-DA), respectively. The Bioconductor R package “DESeq2” (version 1.28.1) was used to identify the significantly different bacterial genus (Benjamini-Hochberg adjusted *p* values < 0.05 and |log2 fold change| > 1.5).

### 4.6. Sera Metabolomics Analysis

#### 4.6.1. Metabolites Extraction for Gas Chromatography-Mass Spectrometry (GC-MS)

An aliquot of 50 μL sera sample was extracted with 200 μL methanol, added to 5 μL L-2-Chlorophenylalanine (1 mg/mL stock in dH2O) as an internal standard, vortex-mixed for 30 s, ultrasound treated for 10 min (incubated in ice water), and centrifuged for 15 min at 12,000 rpm, 4 °C. The 180 μL of supernatant was transferred into new 1.5 mL tubes, and 20 μL from each sample were pooled as quality control (QC) samples. The samples were dried completely in a vacuum concentrator without heating, added to 80 μL Methoxy amination hydrochloride (20 mg/mL in pyridine), incubated for 30 min at 80 °C, added to 100 μL of the BSTFA regent (1% TMCS, v/v), incubated for 1.5 h at 70 °C, followed by the adding of 5 μL FAMEs (in chloroform) to the QC sample when cooling to room temperature. All samples were analyzed by a gas chromatograph system coupled with a Pegasus BT time-of-flight mass spectrometer (GC-TOF-MS).

#### 4.6.2. Metabolites Extraction for Liquid Chromatography Mass Spectrometry (LC-MS)

An aliquot of 50 μL sera sample was extracted with 400 μL of extraction solvent (V methanol: V acetonitrile = 1:1), followed by vortexing for 30 s, then ultrasound treatment for 10 min (incubated in ice water), incubation for 1 h at −20 °C to precipitate proteins, and then centrifugation at 12,000 rpm for 15 min at 4 °C. The supernatant (425 μL) was transferred, dried in a vacuum concentrator without heating, added to 100 μL extraction solvent (V acetonitrile: V water = 1:1), vortexed for 30 s, sonicated for 10 min (4 °C water bath), and then centrifuged for 15 min at 12,000 rpm, 4 °C. The supernatant (60 μL) was transferred into a 2 mL LC/MS glass vial: 10 μL from each sample was pooled as QC sample for the UHPLC-QTOF-MS analysis.

#### 4.6.3. Metabolomics Analysis

The sera metabolites were analyzed by gas chromatography-mass spectrometry (GC-MS) and liquid chromatography-mass spectrometry (LC-MS). An Agilent 7890 gas chromatograph (Agilent Technologies, Wilmington, DE, USA) coupled with a LECO Pegasus BT time-of-flight mass spectrometer (LECO Corporation, St. Joseph, MI, USA) was used for analysis. The MS-DIAL software (RIKEN Center for Sustainable Resource Science, Kanagawa, Japan) [53] and BinBase database system (FiehnLab, Davis, CA, USA) were used for raw peak extraction, baseline data filtration and calibration, peak alignment, deconvolution analysis, peak identification, and peak area integration [54]. For metabolites identification, both mass spectra and retention index matches were considered. We removed peaks detected in ≤50% of the quality control (QC) samples or <50% samples of every group except the QC group or RSD > 30% in QC samples [55]. Meanwhile, the extracted sera samples were analyzed by a UHPLC system (1290, Agilent Technologies) with an ACQUITY UPLC BEH Amide column (1.7 μm, 2.1 mm × 100 mm; Waters Corporation, Milford, MA, USA) coupled to a TripleTOF 6600 Q-TOF, (SCIEX, Framingham, MA, USA). Peak annotation was conducted by the Bioconductor R package CAMERA post-XCMS data processing, and an in-house MS2 database was applied for metabolites identification.

The differentially-expressed metabolites were calculated using the R package “Omu” (version 1.0.4) with a threshold of Benjamini–Hochberg-adjusted p values less than 0.05. The pathway enrichment was analyzed by using MetaboAnalyst 4.0 (https://www.metaboanalyst.ca/ (accessed on 11 August 2020)).

### 4.7. Correlation Analysis

The Correlation between clinical indices, as well as the metabolites and microbiota, were analyzed by the Pearson correlation with the Stats Package from R software (version 4.0.2).

### 4.8. Statistical Analysis

All statistical analyses were performed using the Statistical Package for Social Sciences, version 12.01 (SPSS Inc., Chicago, IL, USA). Continuous variables were expressed as the mean ± standard deviation. The chi-square test or Fisher’s exact test was used to compare categorical variables, while the Mann–Whitney U test was used to compare continuous variables. The Wilcoxon signed-rank test was used to compare baseline and postoperative variables. Friedman’s one-way analysis of variance followed by a post hoc test was used to analyze the differences among plasma levels before surgery, as well as at three, twelve, and twenty-four months after surgery. Correlations between the two groups were examined using Spearman’s correlation method. A *p* value of less than 0.05 was considered statistically significant.

## 5. Conclusions

The main findings reported from this study are briefly described as follows: (1) metabolic surgery ameliorates the clinical syndromes of patients who suffered from obese diabetes and related comorbidities; (2) changes in the microbiome composition and diversity were evident after metabolic surgery; (3) significant changes in some sera metabolites were observed after metabolic surgery; (4) changes in the metabolites were associated with alterations in some bacterial groups. Thus, results obtained in this study support significant ameliorative effects from metabolic surgery and highlight the close interaction between the gut microbiota and carbohydrate metabolism, leading to the improvement of whole-body metabolisms after metabolic surgery. Based on the results obtained in this study, specific bacterial species/strains associated with the metabolic surgery and metabolic ameliorative effects will be selected for subsequent studies. Novel strategy-based characterization of the potential probiotics and their derived metabolites will be developed for the amelioration of diabetes and related syndromes.

## Figures and Tables

**Table 1:** Effect of metabolic surgery on clinical and circulating biomarkers for patients.

	Pre-Surgery	3 Months	12 Months	24 Months
Age (year-old)	42.4 (8.6)			
Gender	Male (*n* = 8); Female (*n* = 5)			
Surgery	Gastric bypass (*n* = 6); Sleeve gastrectomy (*n* = 4); DJB–SG (*n* = 3)			
Weight (kg)	98.0 (15.6)	80.3 (12.3) **	71.1 (13.0) ****	71.2 (12.1) ****
BMI (kg/m^2^)	35.6 (3.2)	29.1 (2.3) ****	25.8 (3.0) ****	26.0 (3.1) ****
Systolic BP (mmHg)	145.8 (13.8)	134.8 (16.5)	132.8 (18.1)	130.8 (20.9)
Diastolic BP (mmHg)	90.0 (12.5)	77.9 (10.1)	79.2 (12.3)	78.9 (17.0)
Total cholesterol (mg/dL)	198.9 (35.3)	171.0 (29.0) *	166.5 (21.7) *	159.6 (20.7) **
HDL (mg/dL)	39.2 (7.3)	37.1 (6.4)	48.9 (9.1) *	50.9 (10.2) **
LDL (mg/dL)	123.7 (27.8)	104.5 (43.0)	100.6 (23.0)	97.1 (20.6)
Triglycerides (mg/dL)	222.9 (173.6)	118.7 (65.4) *	79.25 (31.7) **	85 (35.4) **
Fasting glucose (mg/dL)	136.5 (43.5)	98.9 (20.1) **	96.6 (16.3) **	101.6 (19.2) **
Fasting insulin (mU/L)	41.0 (68.8)	7.7 (3.3)	10.4 (13.8)	11.4 (13.2)
C-peptide (ng/mL)	3.3 (1.5)	2.0 (0.8) **	1.6 (0.5) ***	1.5 (0.6) ***
HbA1c (%)	7.9 (1.4)	5.8 (0.4) ***	6.0 (1.8) **	5.9 (1.0) ***
HOMA-IR	14.3 (24.3)	1.9 (0.8) *	2.8 (4.6)	3.2 (4.1)
Uric acid (mg/dL)	6.7 (1.5)	6.1 (1.0)	5.64 (1.4)	5.9 (1.2)
Creatinine (mg/dL)	0.83 (0.12)	0.80 (0.13)	0.77 (0.12)	0.76 (0.23)
AST (U/L)	25.2 (13.4)	21.9 (9.5)	19.3 (10.6)	20.8 (5.7)
ALT (U/L)	40.0 (25.4)	27.1 (12.4)	28.6 (28.0)	23.2 (11.4)
Albumin (g/dL)	4.5 (0.2)	4.3 (0.2)	5.1 (2.8)	4.4 (0.2)
ALP (U/L)	68.3 (19.6)	73.40 (18.6)	72.91 (18.7)	70.67 (18.0)
γ-GT (U/L)	41.3 (24.6)	22.8 (9.1) **	18.5 (9.2) ***	18.2 (5.9) ***
Acyl-Ghrelin (pg/mL)	40.3 (8.6)	38.1 (7.1)	42.0 (12.9)	37.2 (18.2)
Des-Acyl-Ghrelin (pg/mL)	110.2 (34.4)	47.4 (29.4) **	50.7 (23.6) **	61.4 (34.7) **
GLP-1 (ng/mL)	0.059 (0.036)	0.065 (0.058)	0.086 (0.045)	0.095 (0.044)
PYY (ng/mL)	0.304 (0.121)	0.324 (0.176)	0.392 (0.174)	0.391 (0.118)
GIP (ng/mL)	0.469 (0.126)	0.300 (0.204)	0.386 (0.172)	0.374 (0.216)
FGF-19 (pg/mL)	61.9 (43.3)	115.1 (108.6)	145.7 (130.5)	190.2 (184.0)
FGF-21 (pg/mL)	341.4 (245.3)	413.9 (367.8)	161.7 (104.5)	93.7 (43.3)
FGF-23 (pg/mL)	41.8 (13.7)	78.1 (77.8)	36.2 (8.1)	48.5 (12.4)
Total bile acids (μM)	12.7 (13.4)	7.3 (3.4)	8.4 (9.5)	14.0 (19.7)

DJB–SG: duodeno-jejunal bypass with sleeve gastrectomy; BMI: body mass index; BP: blood pressure; HDL: high-density lipoprotein; LDL: low-density lipoprotein; HbA1C: glycosylated hemoglobin; HOMA-IR: homeostatic model assessment for insulin resistance; AST: aspartate amino transferase; ALT: alanine amino transferase; ALP: alkaline phosphatase; γ-GT: γ-glutamyl transferase; GLP-1: Glucagon-like peptide-1; PYY: Peptide YY; GIP: glucose-dependent insulinotropic peptide; FGF: fibroblast growth factor. Data are presented as mean (standard deviation). Different from pre-surgery: *, *p* < 0.05; **, *p* < 0.01; ***, *p* < 0.005; ****, *p* < 0.001.

## Data Availability

Not applicable.

## References

[1] Tan S.Y., Mei Wong J.L., Sim Y.J., Wong S.S., Mohamed Elhassan S.A., Tan S.H., Ling Lim G.P., Rong Tay N.W., Annan N.C., Bhattamisra S.K. (2019). Type 1 and 2 diabetes mellitus: A review on current treatment approach and gene therapy as potential intervention. Diabetes Metab. Syndr..

[2] Stenberg E., Thorell A. (2020). Insulin resistance in bariatric surgery. Curr. Opin. Clin. Nutr. Metab. Care.

[3] Lee J., Cummings B.P., Martin E., Sharp J.W., Graham J.L., Stanhope K.L., Havel P.J., Raybould H.E. (2012). Glucose sensing by gut endocrine cells and activation of the vagal afferent pathway is impaired in a rodent model of type 2 diabetes mellitus. Am. J. Physiol. Regul. Integr. Comp. Physiol..

[4] Grasset E., Puel A., Charpentier J., Collet X., Christensen J.E., Tercé F., Burcelin R. (2017). A Specific Gut Microbiota Dysbiosis of Type 2 Diabetic Mice Induces GLP-1 Resistance through an Enteric NO-Dependent and Gut-Brain Axis Mechanism. Cell Metab..

[5] Vincent K.M., Sharp J.W., Raybould H.E. (2011). Intestinal glucose-induced calcium-calmodulin kinase signaling in the gut-brain axis in awake rats. Neurogastroenterol. Motil..

[6] Yano J.M., Yu K., Donaldson G.P., Shastri G.G., Ann P., Ma L., Nagler C.R., Ismagilov R.F., Mazmanian S.K., Hsiao E.Y. (2015). Indigenous bacteria from the gut microbiota regulate host serotonin biosynthesis. Cell.

[7] Fournel A., Drougard A., Duparc T., Marlin A., Brierley S.M., Castro J., Le-Gonidec S., Masri B., Colom A., Lucas A. (2017). Apelin targets gut contraction to control glucose metabolism via the brain. Gut.

[8] Michel K., Zeller F., Langer R., Nekarda H., Kruger D., Dover T.J., Brady C.A., Barnes N.M., Schemann M. (2005). Serotonin excites neurons in the human submucous plexus via 5-HT3 receptors. Gastroenterology.

[9] Abot A., Lucas A., Bautzova T., Bessac A., Fournel A., Le-Gonidec S., Valet P., Moro C., Cani P.D., Knauf C. (2018). Galanin enhances systemic glucose metabolism through enteric Nitric Oxide Synthase-expressed neurons. Mol. Metab..

[10] Cleary J.L., Condren A.R., Zink K.E., Sanchez L.M. (2017). Calling all hosts: Bacterial communication in situ. Chem.

[11] Clemente J.C., Ursell L.K., Parfrey L.W., Knight R. (2012). The impact of the gut microbiota on human health: An integrative view. Cell.

[12] Sekirov I., Russell S.L., Antunes L.C., Finlay B.B. (2010). Gut microbiota in health and disease. Physiol. Rev..

[13] Kennedy P.J., Cryan J.F., Dinan T.G., Clarke G. (2014). Irritable bowel syndrome: A microbiome-gut-brain axis disorder?. World J. Gastroenterol..

[14] Mayer E.A. (2000). The neurobiology of stress and gastrointestinal disease. Gut.

[15] Chen C.Y., Fujimiya M., Laviano A., Chang F.Y., Lin H.C., Lee S.D. (2010). Modulation of ingestive behavior and gastrointestinal motility by ghrelin in diabetic animals and humans. J. Chin. Med. Assoc..

[16] Davies N.K., O’Sullivan J.M., Plank L.D., Murphy R. (2019). Altered gut microbiome after bariatric surgery and its association with metabolic benefits: A systematic review. Surg. Obes. Relat. Dis..

[17] Gutiérrez-Repiso C., Moreno-Indias I., de Hollanda A., Martín-Núñez G.M., Vidal J., Tinahones F.J. (2019). Gut microbiota specific signatures are related to the successful rate of bariatric surgery. Am. J. Transl. Res..

[18] Wu J., Zhang P.B., Ren Z.Q., Zhou F., Hu H.H., Zhang H., Xue K.K., Xu P., Shao X.Q. (2019). Changes of serum lipopolysaccharide, inflammatory factors, and cecal microbiota in obese rats with type 2 diabetes induced by Roux-en-Y gastric bypass. Nutrition.

[19] Samczuk P., Luba M., Godzien J., Mastrangelo A., Hady H.R., Dadan J., Barbas C., Gorska M., Kretowski A., Ciborowski M. (2018). “Gear mechanism” of bariatric interventions revealed by untargeted metabolomics. J. Pharm. Biomed. Anal..

[20] Samczuk P., Ciborowski M., Kretowski A. (2018). Application of Metabolomics to Study Effects of Bariatric Surgery. J. Diabetes Res..

[21] Wang W., Cheng Z., Wang Y., Dai Y., Zhang X., Hu S. (2019). Role of Bile Acids in Bariatric Surgery. Front. Physiol..

[22] Huffman K.M., Shah S.H., Stevens R.D., Bain J.R., Muehlbauer M., Slentz C.A., Tanner C.J., Kuchibhatla M., Houmard J.A., Newgard C.B. (2009). Relationships between circulating metabolic intermediates and insulin action in overweight to obese, inactive men and women. Diabetes Care.

[23] Newgard C.B., An J., Bain J.R., Muehlbauer M.J., Stevens R.D., Lien L.F., Haqq A.M., Shah S.H., Arlotto M., Slentz C.A. (2009). A branched-chain amino acid-related metabolic signature that differentiates obese and lean humans and contributes to insulin resistance. Cell Metab..

[24] Osataphan S., Patti M.E. (2019). Trim the gut, lose the weight—and the bone. J. Clin. Investig..

[25] Lee W.J., Chen C.Y., Chong K., Lee Y.C., Chen S.C., Lee S.D. (2011). Changes in postprandial gut hormones after metabolic surgery: A comparison of gastric bypass and sleeve gastrectomy. Surg. Obes. Relat. Dis..

[26] Wang J.W., Chen P.Y., Huang H.H., Yeh C., Chen S.C., Lee W.J., Chen C.Y. (2021). Change of plasma amylin after bariatric surgery challenged by oral glucose is associated with remission of type 2 diabetes mellitus. J. Chin. Med. Assoc..

[27] Chen C.Y., Asakawa A., Fujimiya M., Lee S.D., Inui A. (2009). Ghrelin gene products and the regulation of food intake and gut motility. Pharmacol. Rev..

[28] Chen C.Y., Lee W.J., Asakawa A., Fujitsuka N., Chong K., Chen S.C., Lee S.D., Inui A. (2013). Insulin secretion and interleukin-1β dependent mechanisms in human diabetes remission after metabolic surgery. Curr. Med. Chem..

[29] Russel S.M., Valle V., Spagni G., Hamilton S., Patel T., Abdukadyrov N., Dong Y., Gangemi A. (2020). Physiologic Mechanisms of Type II Diabetes Mellitus Remission Following Bariatric Surgery: A Meta-analysis and Clinical Implications. J. Gastrointest. Surg..

[30] Ben-Porat T., Weiss-Sadan A., Rottenstreich A., Sherf-Dagan S., Schweiger C., Yosef-Levi I.M., Weiner D., Azulay O., Sakran N., Harari R. (2019). Nutritional Management for Chronic Kidney Disease Patients who Undergo Bariatric Surgery: A Narrative Review. Adv. Nutr..

[31] Liu J., Prudom C.E., Nass R., Pezzoli S.S., Oliveri M.C., Johnson M.L., Veldhuis P., Gordon D.A., Howard A.D., Witcher D.R. (2008). Novel ghrelin assays provide evidence for independent regulation of ghrelin acylation and secretion in healthy young men. J. Clin. Endocrinol. Metab..

[32] Chen C.Y., Inui A., Asakawa A., Fujino K., Kato I., Chen C.C., Ueno N., Fujimiya M. (2005). Des-acyl ghrelin acts by CRF type 2 receptors to disrupt fasted stomach motility in conscious rats. Gastroenterology.

[33] Haghighi S., Amini M., Pournaghshband Z., Amini P., Hovsepian S. (2011). Relationship between gamma-glutamyl transferase and glucose intolerance in first degree relatives of type 2 diabetics patients. J. Res. Med. Sci..

[34] Huang H.H., Lee W.J., Chen S.C., Chen T.F., Lee S.D., Chen C.Y. (2019). Bile Acid and Fibroblast Growth Factor 19 Regulation in Obese Diabetics, and Non-Alcoholic Fatty Liver Disease after Sleeve Gastrectomy. J. Clin. Med..

[35] Zachariah P.J., Chen C.Y., Lee W.J., Chen S.C., Ser K.H., Chen J.C., Lee Y.C. (2016). Compared to Sleeve Gastrectomy, Duodenal-Jejunal Bypass with Sleeve Gastrectomy Gives Better Glycemic Control in T2DM Patients, with a Lower β-Cell Response and Similar Appetite Sensations: Mixed-Meal Study. Obes. Surg..

[36] Guyton K., Alverdy J.C. (2017). The gut microbiota and gastrointestinal surgery. Nat. Rev. Gastroenterol. Hepatol..

[37] Ulker İ., Yildiran H. (2019). The effects of bariatric surgery on gut microbiota in patients with obesity: A review of the literature. Biosci. Microbiota Food Health.

[38] Depommier C., Everard A., Druart C., Plovier H., Van Hul M., Vieira-Silva S., Falony G., Raes J., Maiter D., Delzenne N.M. (2019). Supplementation with Akkermansia muciniphila in overweight and obese human volunteers: A proof-of-concept exploratory study. Nat. Med..

[39] Hu R., Zeng F., Wu L., Wan X., Chen Y., Zhang J., Liu B. (2019). Fermented carrot juice attenuates type 2 diabetes by mediating gut microbiota in rats. Food Funct..

[40] Wei X., Tao J., Xiao S., Jiang S., Shang E., Zhu Z., Qian D., Duan J. (2018). Xiexin Tang improves the symptom of type 2 diabetic rats by modulation of the gut microbiota. Sci. Rep..

[41] Hu T.G., Wen P., Shen W.Z., Liu F., Li Q., Li E.N., Liao S.T., Wu H., Zou Y.X. (2019). Effect of 1-Deoxynojirimycin Isolated from Mulberry Leaves on Glucose Metabolism and Gut Microbiota in a Streptozotocin-Induced Diabetic Mouse Model. J. Nat. Prod..

[42] Kang X., Zhan L., Lu X., Song J., Zhong Y., Wang Y., Yang Y., Fan Z., Jiang X., Sun R. (2020). Characteristics of Gastric Microbiota in GK Rats with Spontaneous Diabetes: A Comparative Study. Diabetes Metab. Syndr. Obes..

[43] Felig P. (1973). The glucose-alanine cycle. Metabolism.

[44] Petersen K.F., Dufour S., Cline G.W., Shulman G.I. (2019). Regulation of hepatic mitochondrial oxidation by glucose-alanine cycling during starvation in humans. J. Clin. Investig..

[45] Adeva M., González-Lucán M., Seco M., Donapetry C. (2013). Enzymes involved in l-lactate metabolism in humans. Mitochondrion.

[46] Lee W.J., Hur K.Y., Lakadawala M., Kasama K., Wong S.K., Chen S.C., Lee Y.C., Ser K.H. (2013). Predicting success of metabolic surgery: Age, body mass index, C-peptide, and duration score. Surg. Obes. Relat. Dis..

[47] Huang H.H., Yeh C., Chen J.C., Lee T.H., Chen S.C., Lee W.J., Chen C.Y. (2018). Does bariatric surgery influence plasma levels of fetuin-A and leukocyte cell-derived chemotaxin-2 in patients with type 2 diabetes mellitus?. PeerJ.

[48] Lee W.J., Lee K.T., Kasama K., Seiki Y., Ser K.H., Chun S.C., Chen J.C., Lee Y.C. (2014). Laparoscopic single-anastomosis duodenal-jejunal bypass with sleeve gastrectomy (SADJB-SG): Short-term result and comparison with gastric bypass. Obes. Surg..

[49] Lee W.J., Chong K., Ser K.H., Lee Y.C., Chen S.C., Chen J.C., Tsai M.H., Chuang L.M. (2011). Gastric bypass vs sleeve gastrectomy for type 2 diabetes mellitus: A randomized controlled trial. Arch. Surg..

[50] Nakayama J., Watanabe K., Jiang J., Matsuda K., Chao S.H., Haryono P., La-Ongkham O., Sarwoko M.A., Sujaya I.N., Zhao L. (2015). Diversity in gut bacterial community of school-age children in Asia. Sci. Rep..

[51] Chang C.J., Lin C.S., Lu C.C., Martel J., Ko Y.F., Ojcius D.M., Tseng S.F., Wu T.R., Chen Y.Y., Young J.D. (2015). Ganoderma lucidum reduces obesity in mice by modulating the composition of the gut microbiota. Nat. Commun..

[52] Chang C.J., Lu C.C., Lin C.S., Martel J., Ko Y.F., Ojcius D.M., Wu T.R., Tsai Y.H., Yeh T.S., Lu J.J. (2018). Antrodia cinnamomea reduces obesity and modulates the gut microbiota in high-fat diet-fed mice. Int. J. Obes..

[53] Tsugawa H., Cajka T., Kind T., Ma Y., Higgins B., Ikeda K., Kanazawa M., VanderGheynst J., Fiehn O., Arita M. (2015). MS-DIAL: Data-independent MS/MS deconvolution for comprehensive metabolome analysis. Nat. Methods.

[54] Kind T., Wohlgemuth G., Lee D.Y., Lu Y., Palazoglu M., Shahbaz S., Fiehn O. (2009). FiehnLib: Mass spectral and retention index libraries for metabolomics based on quadrupole and time-of-flight gas chromatography/mass spectrometry. Anal. Chem..

[55] Dunn W.B., Broadhurst D., Begley P., Zelena E., Francis-McIntyre S., Anderson N., Brown M., Knowles J.D., Halsall A., Haselden J.N. (2011). Procedures for large-scale metabolic profiling of serum and plasma using gas chromatography and liquid chromatography coupled to mass spectrometry. Nat. Protoc..

